# Immobile polyanionic backbone enables a 900-μm-thick electrode for compact energy storage with unprecedented areal capacitance

**DOI:** 10.1093/nsr/nwae207

**Published:** 2024-06-14

**Authors:** Haoran Li, Zhitan Wu, Xiaochen Liu, Haotian Lu, Weichao Zhang, Fangbing Li, Hongyuan Yu, Jinyang Yu, Boya Zhang, Zhenxin Xiong, Ying Tao, Quan-Hong Yang

**Affiliations:** Nanoyang Group, Tianjin Key Laboratory of Advanced Carbon and Electrochemical Energy Storage, School of Chemical Engineering and Technology, National Industry-Education Integration Platform of Energy Storage, and Collaborative Innovation Center of Chemical Science and Engineering, Tianjin University, Tianjin 300072, China; Haihe Laboratory of Sustainable Chemical Transformations, Tianjin 300192, China; Nanoyang Group, Tianjin Key Laboratory of Advanced Carbon and Electrochemical Energy Storage, School of Chemical Engineering and Technology, National Industry-Education Integration Platform of Energy Storage, and Collaborative Innovation Center of Chemical Science and Engineering, Tianjin University, Tianjin 300072, China; Haihe Laboratory of Sustainable Chemical Transformations, Tianjin 300192, China; Joint School of the National University of Singapore and Tianjin University, International Campus of Tianjin University, Fuzhou 350207, China; Nanoyang Group, Tianjin Key Laboratory of Advanced Carbon and Electrochemical Energy Storage, School of Chemical Engineering and Technology, National Industry-Education Integration Platform of Energy Storage, and Collaborative Innovation Center of Chemical Science and Engineering, Tianjin University, Tianjin 300072, China; Nanoyang Group, Tianjin Key Laboratory of Advanced Carbon and Electrochemical Energy Storage, School of Chemical Engineering and Technology, National Industry-Education Integration Platform of Energy Storage, and Collaborative Innovation Center of Chemical Science and Engineering, Tianjin University, Tianjin 300072, China; Haihe Laboratory of Sustainable Chemical Transformations, Tianjin 300192, China; Joint School of the National University of Singapore and Tianjin University, International Campus of Tianjin University, Fuzhou 350207, China; Nanoyang Group, Tianjin Key Laboratory of Advanced Carbon and Electrochemical Energy Storage, School of Chemical Engineering and Technology, National Industry-Education Integration Platform of Energy Storage, and Collaborative Innovation Center of Chemical Science and Engineering, Tianjin University, Tianjin 300072, China; Haihe Laboratory of Sustainable Chemical Transformations, Tianjin 300192, China; Nanoyang Group, Tianjin Key Laboratory of Advanced Carbon and Electrochemical Energy Storage, School of Chemical Engineering and Technology, National Industry-Education Integration Platform of Energy Storage, and Collaborative Innovation Center of Chemical Science and Engineering, Tianjin University, Tianjin 300072, China; Haihe Laboratory of Sustainable Chemical Transformations, Tianjin 300192, China; Nanoyang Group, Tianjin Key Laboratory of Advanced Carbon and Electrochemical Energy Storage, School of Chemical Engineering and Technology, National Industry-Education Integration Platform of Energy Storage, and Collaborative Innovation Center of Chemical Science and Engineering, Tianjin University, Tianjin 300072, China; Haihe Laboratory of Sustainable Chemical Transformations, Tianjin 300192, China; Nanoyang Group, Tianjin Key Laboratory of Advanced Carbon and Electrochemical Energy Storage, School of Chemical Engineering and Technology, National Industry-Education Integration Platform of Energy Storage, and Collaborative Innovation Center of Chemical Science and Engineering, Tianjin University, Tianjin 300072, China; Haihe Laboratory of Sustainable Chemical Transformations, Tianjin 300192, China; Nanoyang Group, Tianjin Key Laboratory of Advanced Carbon and Electrochemical Energy Storage, School of Chemical Engineering and Technology, National Industry-Education Integration Platform of Energy Storage, and Collaborative Innovation Center of Chemical Science and Engineering, Tianjin University, Tianjin 300072, China; Haihe Laboratory of Sustainable Chemical Transformations, Tianjin 300192, China; Nanoyang Group, Tianjin Key Laboratory of Advanced Carbon and Electrochemical Energy Storage, School of Chemical Engineering and Technology, National Industry-Education Integration Platform of Energy Storage, and Collaborative Innovation Center of Chemical Science and Engineering, Tianjin University, Tianjin 300072, China; Haihe Laboratory of Sustainable Chemical Transformations, Tianjin 300192, China; Nanoyang Group, Tianjin Key Laboratory of Advanced Carbon and Electrochemical Energy Storage, School of Chemical Engineering and Technology, National Industry-Education Integration Platform of Energy Storage, and Collaborative Innovation Center of Chemical Science and Engineering, Tianjin University, Tianjin 300072, China; Haihe Laboratory of Sustainable Chemical Transformations, Tianjin 300192, China; Nanoyang Group, Tianjin Key Laboratory of Advanced Carbon and Electrochemical Energy Storage, School of Chemical Engineering and Technology, National Industry-Education Integration Platform of Energy Storage, and Collaborative Innovation Center of Chemical Science and Engineering, Tianjin University, Tianjin 300072, China; Haihe Laboratory of Sustainable Chemical Transformations, Tianjin 300192, China; Joint School of the National University of Singapore and Tianjin University, International Campus of Tianjin University, Fuzhou 350207, China

**Keywords:** gel polymer electrolyte, thick electrode, ion transport, areal capacitance, compact energy storage

## Abstract

Thickening of electrodes is crucial for maximizing the proportion of active components and thus improving the energy density of practical energy storage cells. Nevertheless, trade-offs between electrode thickness and electrochemical performance persist because of the considerably increased ion transport resistance of thick electrodes. Herein, we propose accelerating ion transport through thick and dense electrodes by establishing an immobile polyanionic backbone within the electrode pores; and as a proof of concept, gel polyacrylic electrolytes as such a backbone are *in situ* synthesized for supercapacitors. During charge and discharge, protons rapidly hop among RCOO^−^ sites for oriented transport, fundamentally reducing the effects of electrode tortuosity and polarization resulting from concentration gradients. Consequently, nearly constant ion transport resistance per unit thickness is achieved, even in the case of a 900-μm-thick dense electrode, leading to unprecedented areal capacitances of 14.85 F cm^−2^ at 1 mA cm^−2^ and 4.26 F cm^−2^ at 100 mA cm^−2^. This study provides an efficient method for accelerating ion transport through thick and dense electrodes, indicating a significant solution for achieving high energy density in energy storage devices, including but not limited to supercapacitors.

## Teaser

An immobile polyanionic backbone within electrode pores allows for a thickness-independent ion transport behavior, leading to exceptional capacitive energy storage with thick electrodes up to 900 μm.

## INTRODUCTION

Owing to their remarkable rate capability and long life span, supercapacitors are widely used for efficiently storing and delivering electrical energy, particularly at high rates [1–7]. However, current advances are limited by their unsatisfactory energy density [7,8]. Increasing the fraction of active materials in a cell through the fabrication of thick electrodes stands as one of the most direct and efficient strategies for enhancing their energy density, premised on the outstanding ion transport properties [9,10]. In fact, the rapidly growing ion transport resistance severely degrades the energy density of supercapacitors as electrode thickness is further increased, constituting a common bottleneck in the design of practical devices. This is attributed to the dramatically increased tortuosity of electrodes and severe polarization arising from the ion concentration gradient, which is particularly pronounced in the highly dense materials preferred by compact energy storage [11,12].

To utilize thick electrodes, numerous strategies such as pore engineering [13–19] and electrolyte optimization [20–23] have been proposed. For example, 400-μm-thick graphene monolith electrodes have been employed to create supercapacitors with a high volumetric energy density by optimizing pore formation for optimum tortuosity [16]. Moreover, Xia *et al.* configured MXene electrodes with perpendicularly aligned ion transport pathways to achieve thickness-independent rate capabilities as the electrode thickness increased from 40 to 200 μm [19], yet suffering from low packing density and unsatisfactory volumetric capacitance. In addition, electrolyte engineering is crucial for gel or solid-state electrolytes owing to their lower ionic conductivity and inferior interface compatibility compared to liquid electrolytes. Li *et al.* fabricated a 500-μm-thick multi-wall carbon nanotube-based supercapacitor with excellent flexibility and improved interfacial contact between the electrode and electrolyte through bottom-up infiltration of polymerized gel electrolytes [22], resulting in an areal capacitance of 2662 mF cm^−2^ at a scan rate of 2 mV s^−1^. Although the aforementioned advances have successfully reduced the ion transport pathways to enhance the utilization of active materials in thick electrodes, ion transport in these systems primarily relies on individual diffusion, which is significantly influenced by the tortuosity of the electrode. Therefore, the thickening of electrodes is still limited by the commonly known random thermal motion behavior of ions, although it can be accelerated by applying fields. Significant challenges persist in achieving a thickness-independent ion transport behavior within thick and dense electrodes.

Herein, we have developed dense electrodes up to 900 μm thick for symmetric supercapacitors, featuring a dense graphene matrix infilled with immobile polyanionic backbones. The employed gel polyacrylic electrolyte (PAA-based GPE) ensures conformal interfacial contact with electrodes benefiting from its *in-situ* formation and exhibits a high intrinsic ionic conductivity of 0.36 S cm^−1^. Furthermore, the immobile polyacrylic polymer chains act as efficient pathways for directing proton transport and mitigate the effects of concentration polarization. Consequently, almost constant ion transport resistances per unit thickness are achieved by PAA-based GPE when increasing the electrode thickness up to 900 μm (with a mass loading of ∼85 mg cm^−2^). This near millimeter-level electrode delivers an unprecedented areal capacitance of 14.85 F cm^−2^ at 1 mA cm^−2^ and 4.26 F cm^−2^ at 100 mA cm^−2^. Using *in situ* attenuated total reflectance-surface enhanced infrared absorption spectroscopy (ATR-SEIRAS), electrochemical impedance spectroscopy (EIS), and finite element method (FEM) simulation, we show that the accelerated ion transport with PAA-based GPE mainly arises from the rapid hopping of protons through RCOO^−^ sites on polyacrylic chains under an electric field, which completes a long-range diffusion of protons through the formation and breakage of RCOO-H bonds. This study provides new insights into the driving force behind ion transport and presents an alternative approach to utilizing thick electrodes in energy storage devices for achieving high energy densities, not limited to supercapacitors.

## RESULTS AND DISCUSSION

**Figure 1.**
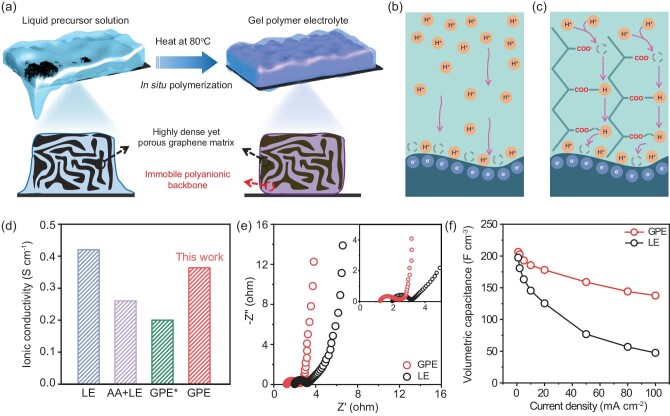
*In situ* formation of PAA-based GPE and its proton transport behavior within HPGM-based electrodes. (a) Schematics illustrating the fabrication process of dense and thick electrodes infilled with PAA-based GPE by *in situ* polymerization of a liquid precursor solution. Schematics of the proton transport process of (b) LE and (c) PAA-based GPE. (d) The ionic conductivity of LE, AA + LE, GPE* and PAA-based GPE (GPE* represents PAA-based GPE without ZnSO_4_). (e) EIS, and (f) rate capability of HPGM electrodes with LE and PAA-based GPE. The thickness of the electrode is ∼20 μm, with a mass loading of ∼2 mg cm^−2^.

A highly dense yet porous graphene monolith (HPGM), obtained by capillary-induced drying of graphene hydrogels, was chosen as the model electrode material due to its high volumetric capacitance [24]. The precursor solution for the gel polymer electrolyte (GPE) was prepared by mixing monomers (acrylic acid, AA), crosslinkers (ZnSO_4_ and *N,N*′-methylene bisacrylamide, MBAA), and initiators (ammonium persulfate, APS) in the liquid electrolyte (LE) of 1 M H_2_SO_4_. This solution was then thoroughly wetted into two HPGM-based electrodes before polymerization in a sealed soft pack to form polyacrylic backbones within the electrodes (Fig. 1a, see Materials and Methods for details). This device fabrication process is almost the same as that using conventional LEs, except for the addition of precursors of polyacrylic backbones and heating processes. The contact angle (44.9°) of the precursor solution on the calendared HPGM-based electrode is smaller than that of the LE (110.6°), suggesting better surface wettability (Fig. S1) and ensuring conformal contact between the electrode and electrolyte. Scanning electron microscopy (SEM) shows that the electrodes feature a smooth surface, indicating that the pores of electrodes are completely filled with GPE (Figs S2 and S3).

Unlike conventional LEs where protons diffuse over long distances (Fig. 1b), the proton transport near the interface between an electrode and PAA-based GPE may follow a ‘proton hopping’ manner, also known as ‘Grotthuss mechanism’, due to the enrichment of hydrogen bonding [25,26]. This mechanism involves the forming and breaking of RCOO-H bonds and the oriented migration of protons among the polymer chains when applying an electric field (Fig. 1c). We first investigate the properties of GPE formed solely from the precursor solution, which appears as a transparent gel in a quasi-solid state after polymerization (Fig. S4). The ionic conductivities of electrolytes were assessed by EIS analysis using a Swagelok cell (Fig. S5). As shown in Fig. 1d, the ionic conductivity of LE decreases from 0.43 to 0.25 S cm^−1^ when AA monomers are added, which further decreases to 0.2 S cm^−1^ after polymerization. However, when ZnSO_4_ is incorporated into the GPE, the resultant material has an ionic conductivity of 0.36 S cm^−1^, which is close to LE and higher than other gel electrolytes such as PVA and PAM (0.08 and 0.13 S cm^−1^) [27,28]. This enhancement is attributed to the establishment of a Zn^2+^-aided double cross-linked network, contributing to improved ion transport properties [29].

In addition, the PAA-based GPE, effectively filling voids in the electrode, binds the electrode together and further reinforces its mechanical properties. Considering the limited flexibility of thick and dense electrodes, a flexible carbon nanotube film was used as a substitute for HPGM to illustrate the mechanical properties of the GPE-incorporated electrodes. Figure S6 shows that carbon nanotube films soaked only in LE exhibit microcracks after folding, while those infused with the PAA-based GPE remain crack-free even after multiple folding cycles. It is evident that the GPE effectively preserves the integrity of the electrode, mitigating the risk of increased series resistance and deteriorated electrochemical performance associated with microcracks. To verify the interaction between the *in situ* formed PAA-based GPE and the electrodes, X-ray photoelectron spectroscopy (XPS) measurements were conducted on the electrodes. As shown in Fig. S7, the carbonyl and carboxyl peaks at 288.5 eV and 289.3 eV of the electrode filled with the gel electrolyte remain after removing the PAA with a hot Na_2_CO_3_ solution at 60°C. This observation indicates a strong interaction at the electrode/electrolyte interface between PAA-based GPE and HPGM.

To investigate the difference in ion transport behavior between electrodes in LE and GPE, we conducted EIS measurements on 20-µm-thick HPGM-based electrodes using a symmetric supercapacitor configuration. The Nyquist plots revealed distinctive features indicating a nonfaradaic process: a semicircle, a straight line inclined at 45° to the real axis, and a quasi-vertical line at frequencies below 0.5 Hz. In both electrolytes, the electrodes have low ohmic resistance (R_ohm_) observed from their small intersections with the real axis, as shown in Fig. 1e. Furthermore, in the mid-frequency range of the Nyquist plot, a clear contrast in ion transport behavior emerged between the two electrolytes. While electrodes with LE had an obvious 45° Warburg-type impedance, those with PAA-based GPE are considerably shorter, almost vertical lines across all frequencies, signifying more efficient ion transport. These findings strongly suggest that the use of PAA-based GPE substantially reduces ion transport resistance. The CV curve of GPE has a more rectangular shape and a higher integral area compared to that of LE at a scan rate of 100 mV s^−1^ (Fig. S8), indicating superior capacitive performance and decreased ion transport resistance, which is consistent with the EIS results. The difference becomes more evident as the scan rate increases (Fig. S9). As shown in Fig. 1f and Fig. S10, a supercapacitor with PAA-based GPE has a specific volumetric capacitance of 206 F cm^−3^ and a gravimetric capacitance of 202 F g^−1^ at a current density of 1 mA cm^−2^, which is almost the same as that of supercapacitors using LE. As current densities increase to 100 mA cm^−2^, supercapacitors with PAA-based GPE maintain a significant capacitance retention of approximately 70% (138 F cm^−3^), which is four times the value of those with LE.

**Figure 2.**
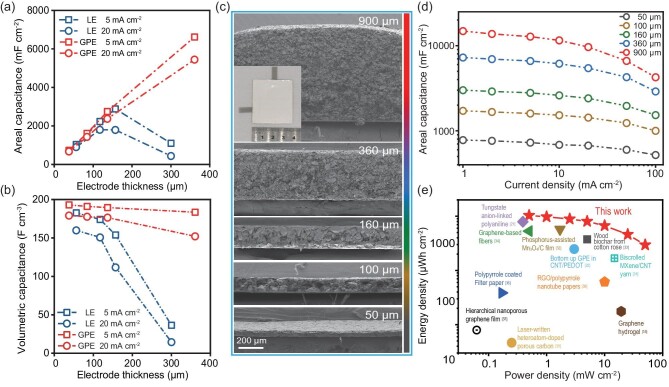
Electrochemical performance of thick HPGM-based electrodes with PAA-based GPE. (a) Areal capacitance and (b) volumetric capacitance of HPGM electrodes with different thicknesses using LE and PAA-based GPE. (c) SEM images of the HPGM electrodes with different thicknesses. The inset shows a digital photo of an assembled supercapacitor using a flexible package with PAA-based GPE and 900 μm electrodes. (d) Rate capabilities of HPGM electrodes with different thicknesses using PAA-based GPE. (e) Areal Ragone plot (per cm^2^ of the SC devices). This work refers to supercapacitors with an electrode thickness of 900 μm tested over potential windows of 0 to 1.0 V [21,22,30–38].

Electrochemical performance tests were conducted on supercapacitors with thick electrodes to demonstrate the practicality of PAA-based GPE to accelerate ion transport. Given the difficulty in achieving precise mass control over the preparation of thick electrodes, electrodes with a similar thickness were used for comparison in subsequent tests. As shown in Fig. 2a, at current densities of 5 mA cm^−2^ and 20 mA cm^−2^, the relationship between areal capacitance and electrode thickness of PAA-based GPE has a nearly linear behavior. When the electrode thickness is 50 μm, the areal capacitance decreases from 728 to 672 mF cm^−2^ as the current density increases from 5 to 20 mA cm^−2^ (a 92% retention). With an increase in thickness to 360 μm for the same change in current density, the capacitance decreases from 6616 to 5444 mF cm^−2^ (an 82% retention). In contrast, the areal capacitance of electrodes using LE at a current density of 5 mA cm^−2^ increases with thickness only up to 160 μm and then decreases significantly. This critical thickness is even lower for a current density of 20 mA cm^−2^. Electrodes with GPE sustained a high volumetric capacitance (from 192 to 183 F cm^−3^ at 5 mA cm^−2^ and from 178 to 152 F cm^−3^ at 20 mA cm^−2^) as the electrode thickness increased from 50 to 360 μm (Fig. 2b). Conversely, the volumetric capacitance of electrodes with LE exhibited a significant decrease as the electrode thickness increased, which is ascribed to the limited utilization of the active materials. For example, at a thickness of 300 μm, the volumetric capacitance of an electrode with LE was only 36 F cm^−3^ at 5 mA cm^−2^, indicating an 80% reduction compared to one 50 μm thick. Furthermore, electrodes with PAA-based GPE demonstrated higher gravimetric capacitance compared to those with LE (Fig. S11). The thickness of the electrodes was determined from their cross-sectional SEM images (Fig. 2c). Figure 2d also shows the corresponding rate capabilities of these electrodes in symmetric supercapacitors using PAA-based GPE, with current densities ranging from 1 mA cm^−2^ to 100 mA cm^−2^. The areal capacitance retention for a 50-μm-thick electrode was 66.7%. At an ultra-high thickness up to 900 μm, the areal capacitance peaked at 14.85 F cm^−2^ under 1 mA cm^−2^, which is the highest value among electric double layer capacitors (Fig. S12, Table S2). Remarkably, it still maintained 4.26 F cm^−2^ at 100 mA cm^−2^, while supercapacitors with LE had almost no capacitance at an electrode thickness of 300 μm (Fig. S13). Further increasing the thickness of dense electrodes to millimeter-level is expected to achieve higher areal capacitance through the utilization of this polyacrylic backbone. However, it is currently constrained by the electrode preparation techniques, such as deformation of the current collector.

When supercapacitors with PAA-based GPE and 900-μm-thick electrodes were subjected to current densities ranging from 1 mA cm^−2^ to 100 mA cm^−2^ in a potential window of 0 to 1.0 V, symmetric and triangular shapes of the galvanostatic charge-discharge (GCD) curves were observed (Fig. S14). Correspondingly, the areal energy density and power density change from 1031 μWh cm^−2^ and 500 μW cm^−2^ to 296 μWh cm^−2^ and 50 mW cm^−2^. These results surpass that of previously reported devices with thick electrodes as depicted in the Ragone plot (Fig. 2e, Table S3), including 3D-printed carbonaceous supercapacitors that provided 0.72 mWh cm^−2^ at a power density of 1.3 mW cm^−2^ [39] and supercapacitors using 800-μm-thick high-phosphorus-doped carbon electrodes that provided 942 μWh cm^−2^ at a power density of 0.6 mW cm^−2^ [13]. These findings highlight the ability of the PAA-based GPE to facilitate rapid ion transport in the thick and dense electrodes prepared from HPGM. In addition, supercapacitors using PAA-based GPE have remarkable cycling stability, retaining 88% and 91% of their initial capacitance after 30 000 cycles using 100-μm-thick and 50-μm-thick electrodes, respectively (Fig. S15).

**Figure 3.**
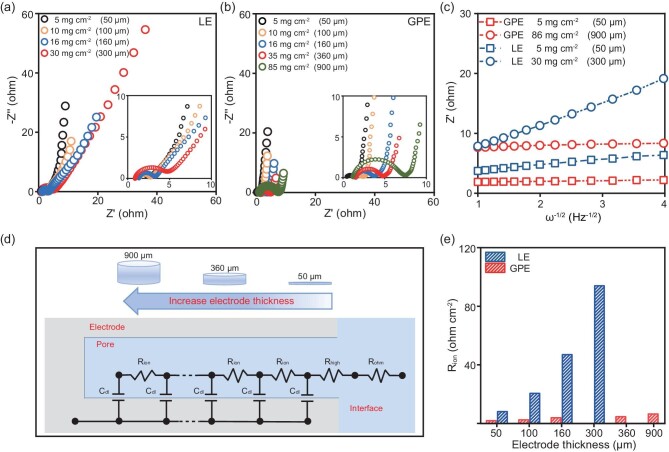
Influence of electrode thickness on the proton transport behavior in LE and PAA-based GPE. Nyquist plots of supercapacitors assembled with electrodes of different thicknesses using (a) LE and (b) PAA-based GPE. (c) Relationship between Z′ and ω^−1/2^ in the low-frequency region. (d) Transmission line equivalent circuit of porous electrodes with different thicknesses. (e) The ion transport impedance of supercapacitors with different electrode thicknesses using LE and PAA-based GPE.

Nyquist plots of individual electrodes with different thicknesses using LE and GPE are shown in Fig. 3a, b. In supercapacitors with PAA-based GPE, the length of the 45° lines is negligible, whereas in supercapacitors with LE, the length of these lines increases steadily as the thickness of electrodes increases. This result implies that the increase in electrode thickness has minimal impact on the rate of ion transport in the supercapacitors when PAA-based GPE is used. The Warburg coefficients (*k*_w_), representing the kinetics of ion diffusion [40], are calculated by linear fitting of the slope obtained from the relationship between Z′ and ω^−1/2^ in the low-frequency range (Fig. 3c). Subsequently, the diffusion coefficients of protons at 25°C were calculated to be 3.13 × 10^−7^ cm^2^ s^−1^ and 6.45 × 10^−10^ cm^2^ s^−1^ for supercapacitors with GPE and LE, respectively (see Supporting Information for the calculation details). The supercapacitors with PAA-based GPEs had lower slopes than those with LE, indicating better ion diffusion kinetics. These results are ascribed to the lower ion transport resistance of the former, which helps achieve rapid charge and discharge.

Figure 3d shows the use of the transmission line model (TLM) for interpreting processes in the porous electrode. In this equivalent circuit, R_ohm_ represents the resistance associated with the electrolyte solution and separator, R_high_ mainly represents the resistance of the electrode-electrolyte interface, and R_ion_ signifies ion transport within the electrode pores. According to the TLM theory for cylindrical pores, R_ion_ is three times the projection of the 45° line onto the Z′ axis [41–43], which is a critical parameter for evaluating the rate capability of the porous electrode throughout charge and discharge. Simulations enable us to obtain the values of R_ohm_, R_high_, and R_ion_ for supercapacitors with different mass loadings and electrolytes (Fig. S16 and Table S4). The R_ion_ of supercapacitors with LE increases to 93.94 Ω as the electrode thickness reaches 300 μm, whereas the value of supercapacitors with PAA-based GPE remains at 6.38 Ω even when the thickness reaches 900 μm (Fig. 3e). These findings suggest that the ion transport resistance of the electrode with PAA-based GPE remains low despite increases in thickness.

**Figure 4.**
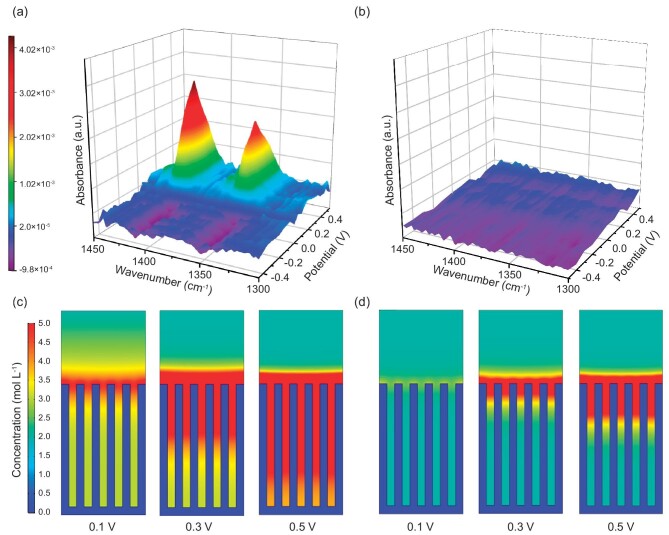
Mechanism investigations. *In situ* ATR-SEIRAS spectra of HPGM electrodes with (a) PAA-based GPE and (b) LE. Proton concentration distributions of porous electrodes with 300 μm thickness at different potentials using (c) PAA-based GPE and (d) LE obtained by FEM simulations.

We used *in situ* ATR-SEIRAS to monitor the changes in functional groups at the surface of the HPGM electrodes when exposed to different electrolytes. The spectra were collected from −0.5 to 0.5 V vs. an Ag/AgCl reference electrode in saturated KCl solution (see detailed method in the Supporting Information). Significantly, the *in situ* ATR-SEIRAS detection range extends ∼10 nm beyond the electrode surface, a dimension comparable to the thickness of the electric double layer (EDL). The vibrational bands, which are centered at ∼1360 cm^−1^ and 1420 cm^−1^ [44,45] and are indicative of -COO^−^ groups, are observed between 0 and 0.5 V (Fig. 4a). The intensity of these vibrational bands gradually rises as the potential increases (Fig. S17). In contrast, no obvious vibrational bands are observed at the surface of HPGM electrode with LE, which indicates that the RCOO^−^ is derived from the ionization of PAA rather than the electrodes themselves (Fig. 4b). Echoing the above-mentioned ‘proton hopping’ mechanism, the correlation between changes in functional groups at the surface of the electrode with PAA-based GPE and electrochemical processes is elucidated as follows: PAA on the electrode surface undergoes ionization into free-moving protons and immobile polyanionic chains under an electric field. The RCOO^−^ groups appear on the electrode surface as protons migrate away from the electrode surface EDL during the application of a positive potential. The absence of vibrational bands at a negative potential is attributed to the migration of protons to the surface of the electrode when the potential falls below 0 V, leading to the formation of RCOOH instead of RCOO^−^. These results provide evidence that the RCOO^−^ sites are active for capturing protons, and the oriented transport of protons is completed by the forming and breaking of RCOO-H bonds under an electric field, appearing as proton hopping through the polyacrylic chains. As shown in Fig. S18, the precursor solution has a slightly higher pH value compared to that of 1 M H_2_SO_4_, indicating a lower proton concentration in the precursor solution. Moreover, the electrode with PAA-based GPE exhibits better electrochemical properties than that with the precursor solution (Fig. S19), demonstrating the necessity to construct polymer chains for orienting proton transport.

Based on the Nernst-Planck-Poisson model, we developed two-dimensional discrete dynamic models and carried out finite element method (FEM) simulations to investigate the difference between proton transport in porous electrodes with LE and GPE. Except for considering electromigration and proton transport processes, the PAA-based GPE case additionally accounts for ionization and proton transport processes through a fixed RCOOH group, whereas LE does not. Applying a potential changing at a rate of 0.05 V s^−1^ to the electrodes, ion transport speeds in different electrolytes were simulated by monitoring ion concentrations at 0.1, 0.3, and 0.5 V. Using COMSOL Multiphysics software, the ion concentration distribution was calculated in a porous electrode with a thickness of 300 μm and a pore size of 20 μm, as illustrated in Fig. 4c and d. The concentration of protons inside the porous electrode with PAA-based GPE is significantly higher than that with LE after an equivalent relaxation period, indicating a more rapid proton transport in GPE. The relationship between current density and potential on a two-dimensional scale was determined by analyzing the charge density of the electrode (Fig. S20). Due to the accelerated proton transport from the electrolyte to the electrode surface, the thick electrode with PAA-based GPE has a higher current density than that with LE. The multi-physics simulation indicates that proton transport is faster in PAA-based GPE, because of an improvement of the sluggish ion transport kinetics in thick electrodes. Overall, *in situ* polymerization of the precursor solution produces a PAA-based GPE that facilitates contact with the electrode and the transport of protons through RCOO^−^ groups immobilized on the polymer chains. Benefitting from these factors, ion transport within the dense graphene networks is accelerated, thus contributing to the efficient use of the thick and dense electrode.

## CONCLUSION

This study demonstrates that the *in situ* polymerization of polyacrylic-based gel polymer electrolytes within the electrodes fundamentally increases ion transport through a thick electrode. The introduced immobile polyanionic backbones serve as directed ion transport pathways where protons hop between RCOO^−^ groups, minimizing influences from the tortuous structure of electrodes and polarization caused by concentration gradients. As a result, the areal capacitance of a 900-μm-thick electrode with PAA-based GPE reaches 14.85 F cm^−2^ at 1 mA cm^−2^, corresponding to an areal energy density of 1096 μWh cm^−2^ at 0.5 mW cm^−2^ based on a supercapacitor, ranking among the highest reported values for electric double layer capacitors. The phenomenon of proton hopping through the fixed RCOO^−^ groups on polyacrylic chains was revealed by *in situ* ATR-SEIRAS and FEM simulations. We believe this work will contribute a final solution to guaranteeing fast ion transport through dense electrodes and address the challenges associated with thick electrodes for practical devices with high energy density.

## MATERIALS AND METHODS

### Materials

All reagents and materials in this work are commercially available and were used without further purification. The acrylic acid (AA) monomers were purchased from Alfa Aesar. Zinc sulfate heptahydrate (ZnSO_4_·7H_2_O), carbon nanotube (CNT) and ammonium persulfate (APS) were purchased from Aladdin. *N,N*′-methylene bisacrylamide (MBAA) was purchased from Heowns. The polytetrafluoroethylene (PTFE) and Super P were purchased from Guangdong Canrd New Energy Technology Co., Ltd.

### Preparation of HPGM

Graphite oxide was prepared from graphite powder using a modified Hummers method as reported previously [46]. The high-density porous graphene macroform (HPGM) was prepared using the method reported in our previous work [24]. The preparation process was as follows: 160 mg graphite oxide was put in 80 mL deionized water and the mixture was ultrasonicated for 2 h to obtain the graphene oxide colloidal suspension (2 mg mL^−1^). The suspension was subjected to a mild centrifugation (4000 rpm for 20 min) to remove thick layers. Then 80 mL of the graphene oxide suspension was placed in a 100-mL Teflon-lined autoclave and exposed to hydrothermal treatment in an anti-explosion oven at 180°C for 6 h to prepare a hydrogel, which was rinsed and then dried for 72 h at 80°C in an air-circulating oven to produce HPGM.

### Assembly of the symmetric supercapacitors with *in situ* formed PAA-based GPE

The HPGM was first pulverized to fine powder, it was then mixed with carbon black and PTFE in a weight ratio of 8:1:1 in an ethanol solution to form a uniform slurry, which was kneaded into a paste and spread uniformly. Then the electrodes were obtained using a 10 mm diameter punch, and were pressed at 10 MPa onto a stainless-steel mesh. A precursor solution of PAA-based GPE was fabricated by dissolving 5.6 mL AA into 40 mL 1 M H_2_SO_4_, followed by adding 7 mg MBAA and 58.6 mg ZnSO_4_·7H_2_O as crosslinkers and 30 mg APS as initiators under stirring. The electrodes and non-woven fabric used as the separator were soaked in the precursor solution for 1 h for later use. Two working electrodes with the separator between them were assembled in a sandwich configuration, which was encapsulated in a flexible package (4 × 4.5 cm^2^); 300 μL of precursor solution was used as an electrolyte during the assembly of symmetric supercapacitors. The *in situ* polymerization of the PAA-based GPE was achieved by heating it at 70°C for 30 min and then keeping it at room temperature for 4 h.

## Supplementary Material

nwae207_Supplemental_File
