# Supplementary material for: Immobile polyanionic backbone enables a 900-μm-thick electrode for compact energy storage with unprecedented areal capacitance
Source: Natl Sci Rev. 2024 Jun 14;11(8):nwae207. doi: 10.1093/nsr/nwae207 (PMC11242447; doi:10.1093/nsr/nwae207)
Supplement: nwae207_Supplemental_File [file nwae207_supplemental_file.pdf]

Immobile polyanionic backbone enables a 900-μm-thick electrode for compact energy storage with unprecedented areal capacitance

Haoran Li[1,2†], Zhitan Wu[1,2,3†], Xiaochen Liu[1†], Haotian Lu[1,2,3], Weichao Zhang[1,2], Fangbing Li[1,2], Hongyuan Yu[1,2], Jinyang Yu[1,2], Boya Zhang[1,2], Zhenxin Xiong[1,2], Ying Tao[1,2*] and Quan-Hong Yang[1,2,3*]

[1]Nanoyang Group, Tianjin Key Laboratory of Advanced Carbon and Electrochemical Energy Storage, School of Chemical Engineering and Technology, National Industry-Education Integration Platform of Energy Storage, and Collaborative Innovation Center of Chemical Science and Engineering, Tianjin University, Tianjin 300072, China

[2]Haihe Laboratory of Sustainable Chemical Transformations, Tianjin 300192, China

[3]Joint School of the National University of Singapore and Tianjin University, International Campus of Tianjin University, Fuzhou 350207, China

† These authors contributed equally to this work.

*E-mail: yingtao@tju.edu.cn, qhyangcn@tju.edu.cn

Methods

Preparation of flexible carbon nanotube (CNT) films

First, the CNTs were dispersed in ethanol and subjected to 30 minutes of sonication to achieve a uniform dispersion with a concentration of 0.5 mg mL^{-1}. Then, the dispersion underwent vacuum filtration through a polypropylene membrane, followed by vacuum drying at room temperature to produce the CNT films. Subsequently, the dried films were cut and immersed in a liquid electrolyte (1 M H_2SO_4) and a precursor solution of PAA-based GPE for 30 min, respectively. Finally, these films were placed onto a PTFE substrate and dried at 80 °C, resulting in the production of two distinct electrolyte-treated CNT films.

Characterization

Scanning electron microscope (SEM) observations were performed on a Regulus 8100 (Hitachi, Japan) with an accelerating voltage of 3.0 kV. The thickness of electrodes was determined by cross-sectional SEM images of three independent samples, and the average value was used to calculate the bulk density. X-ray photoelectron spectroscopy (XPS, Thermo Fisher Scientific ESCALAB Xi+, Al Kα radiation, hv = 1486.6 eV, UK) was used to determine the surface composition.

Electrochemical measurements

The cyclic voltammetry (CV) and galvanostatic charge/discharge (GCD) were conducted using an electrochemical workstation (BioLogic, France) at room temperature. The electrochemical performance of a single electrode was evaluated in a Swagelok cell, with PAA/H_2SO_4 GPE and 1 M H_2SO_4 as electrolytes. The electrochemical impedance spectroscopy (EIS) tests were performed using an electrochemical workstation (Autolab 128N, Switzerland). The cycling stability tests were conducted using a battery test system (Neware BTS-4000, Shenzhen, China). The CV measurements

of the electrode were conducted between 0 and 1 V. The EIS test was performed in the frequency range from 100 kHz to 0.01 Hz at open circuit potential by applying a potential amplitude of 5 mV. Except for the EIS test, all the electrochemical tests were conducted after initially pre-cycling the cells at 20 mV s^{-1} for 10 cycles.

Calculations

The ionic conductivity of the GPE was determined from EIS results, based on the Swagelok cell LiTshown in **Fig. S5**. For the LEs depicted in **Fig. 1b**, their ionic conductivity were determined by introducing an aqueous solution into the testing instrument. In contrast, the ionic conductivities of GPEs were determined by introducing the precursor solution into the instrument and then subjecting it to heat to induce polymerization. The electrolyte resistance was determined from the intercept of the Nyquist plot, and the ionic conductivity was computed using the following equation.

$$\sigma = \frac{L}{SR} \tag{1}$$

where L is the thickness of GPE film (cm), S is the area of stainless steel (cm^2), R is the resistance of GPE (Ω).

The areal specific capacitance (C_a, mF cm^{-2}) of the electrode was calculated from the discharge portion of the GCD curves using the following formula:

$$C_a = \frac{2I\Delta t}{S\Delta V} \tag{2}$$

where I is the discharge current (mA), Δt is the discharge time (s), S (cm^2) is the area of the electrode and ΔV is the discharge voltage range (V).

The areal specific energy density (E_a, mWh cm^{-2}) and power density (P_a, mW cm^{-2}) of the symmetric supercapacitor were calculated according to the following formulae:

$$E_a = \frac{C_a \Delta V^2}{2\times 3600} \tag{3}$$

$$P_a = \frac{E_a \times 3600}{\Delta t} \tag{4}$$

where C_a is the areal capacitance based on symmetric supercapacitor (mF cm^{-2}), ΔV is the discharge voltage range (V), Δt is the discharge time (s).

The diffusion coefficient (D cm^2 s^{-1}) of protons in the electrode with PAA-based GPE and LE were calculated according to the following formula:

$$D = \left(\frac{RT}{n^2 F^2 A\sqrt{2} k_w C^*}\right)^2 \tag{5}$$

where R is the gas constant (J $kg^{-1} \cdot K^{-1}$), T is the temperature(K), n is the charge transfer number, A is the areal of the electrode surface (m^2), kw is the Warburg coefficient (Ω cm^2 mol^{-1}), and C* is the ionic concentration (mol L^{-1}).

***In situ* attenuated-total-reflection surface-enhanced infrared absorption spectroscopy (*In situ* ATR-SEIRAS) test**

Before the *in situ* ATR-SEIRAS test, the reactor was washed with 1.0 M hydrochloric acid solution (HCl) and ethanol several times to remove any possible contaminants. For the working electrode, 30 μL HPGM dispersion (3 mg mL^{-1}) was dropped onto a Si crystal and dried in air. The precursor solution was then dropped on it three times and *in situ* polymerized by heating. A Au film was chemically deposited on the surface of the Si crystal to increase the signal according to the method reported by Miyake et al.[1] The Ag/AgCl soaked in saturated KCl and a glass carbon electrode were used as reference and counter electrode, respectively. All spectra were recorded on Nicolet iS50 FT-IR spectrometer, with a resolution of 4 cm^{-1}.The background was collected in 1.0 M H_2SO_4 before applying each potential. All electrochemical experiments were recorded with a potentiostat (CompactStat.e20250, IVIUM).

Finite-element simulation of ionic mass transfer in the liquid and polyanion gel electrolytes

A two-dimensional discrete model was constructed to simulate ionic mass transfer and investigate the dynamics of the electric double layer in the liquid and polyanion gel electrolytes. The model was based on the Poisson-Nernst-Planck equation and the mechanism of line scan voltammetry measurement. In liquid electrolytes, there are mass balance and charge conservation:

$$\nabla(\varepsilon_0\varepsilon_r\nabla\psi) = -F(z_{H^+}c_{H^+} + z_{SO_4^{2-}}c_{SO_4^{2-}}) \tag{1}$$

$$\frac{\partial c_{H^+}}{\partial t} = -\nabla N_{H^+} = \nabla(D_{H^+}\nabla c_{H^+} + \frac{D_{H^+}Fz_{H^+}c_{H^+}}{RT}\nabla\psi) \tag{2}$$

$$\frac{\partial c_{SO_4^{2-}}}{\partial t} = -\nabla N_{SO_4^{2-}} = \nabla(D_{SO_4^{2-}}\nabla c_{SO_4^{2-}} + \frac{D_{SO_4^{2-}}Fz_{SO_4^{2-}}c_{SO_4^{2-}}}{RT}\nabla\psi) \tag{3}$$

In polyanion gel electrolytes, there are mass balance and charge conservation:

$$\nabla(\varepsilon_0\varepsilon_r\nabla\psi) = -F(z_{H^+}c_{H^+} + z_{SO_4^{2-}}c_{SO_4^{2-}} + z_{ROO^-}c_{ROO^-}) \tag{4}$$

$$\frac{\partial c_{H^+}}{\partial t} = -\nabla N_{H^+} = \nabla(D_{H^+}\nabla c_{H^+} + \frac{D_{H^+}Fz_{H^+}c_{H^+}}{RT}\nabla\psi) \tag{5}$$

$$\frac{\partial c_{SO_4^{2-}}}{\partial t} = -\nabla N_{SO_4^{2-}} = \nabla(D_{SO_4^{2-}}\nabla c_{SO_4^{2-}} + \frac{D_{SO_4^{2-}}Fz_{SO_4^{2-}}c_{SO_4^{2-}}}{RT}\nabla\psi) \tag{6}$$

$$\frac{\partial c_{ROO^-}}{\partial t} = -\nabla N_{ROO^-} = 0\ \mathrm{mol\ L^{-1}s^{-1}} \tag{7}$$

In carbon electrodes, there is charge conservation:

$$\nabla(\sigma_c\nabla\psi) = 0 \tag{8}$$

At the interface between the electrode and electrolyte, the ionic flux is zero due to the absence of Faradaic reactions. Meanwhile, the ion concentration is assumed to remain constant away from the electrolyte-electrode interface. To simulate a line scan voltammetry measurement, the potential of the

carbon electrode is set using the following equation:

$$\psi = \psi_{max} - v * t \tag{9}$$

All finite-element simulations were solved in COMSOL Multiphysics 6.1. The thickness of the porous electrode was set to 300 μm. The simulation parameters were set to be consistent with the electrochemical measurement conditions (**Table S1**).

Table S1. Simulation parameters for finite-element simulation.

Parameter	Symbol	Value
Ion valence of H^+	z_{H^+}	1
Ion valence of SO_4^{2-}	$z_{SO_4^{2-}}$	-2
Valence of ROO^-	z_{ROO^-}	-1
Diffusion coefficient of H^+ in water	D_{H^+}	9.31×10^{-9} m^2 s^{-1}
Diffusion coefficient of SO_4^{2-} in water	$D_{SO_4^{2-}}$	1.96×10^{-9} m^2 s^{-1}
Diffusion coefficient of ROO^-	D_{ROO^-}	0 m^2 s^{-1}
Dielectric coefficient in water at zero electric field	ε_r	78.5
Electrode conductivity	σ_C	2×10^2 S m^{-1}
Bulk concentration of H^+	c_{H^+}	2 mol L^{-1}
Bulk concentration of SO_4^{2-}	$c_{SO_4^{2-}}$	1 mol L^{-1}
The negative charge density of ROO^-	c_{Roo^-}	1 mol L^{-1}
Maximum electrode potential	ψ_{max}	0 V
Minimum electrode potential	ψ_{min}	-0.5 V
Potential scan rate	v	0.05 V s^{-1}

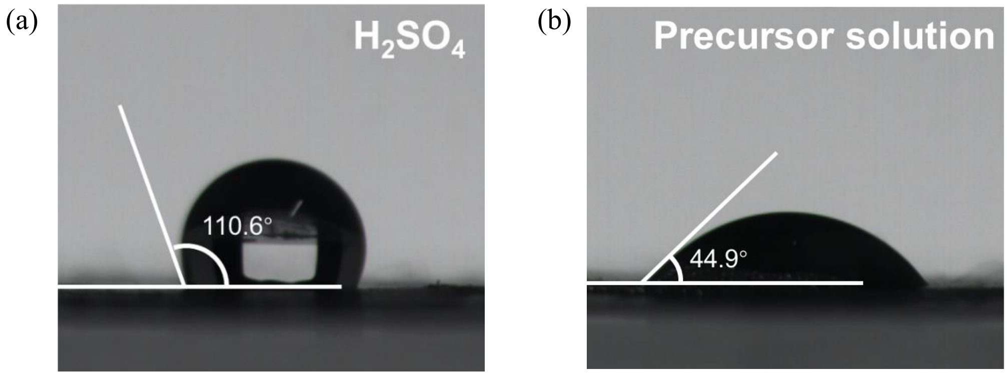

Fig. S1. Images of contact angles between the HPGM electrodes and different electrolytes. (a) 1 M H_2SO_4; (b) precursor solution of PAA-based GPE.

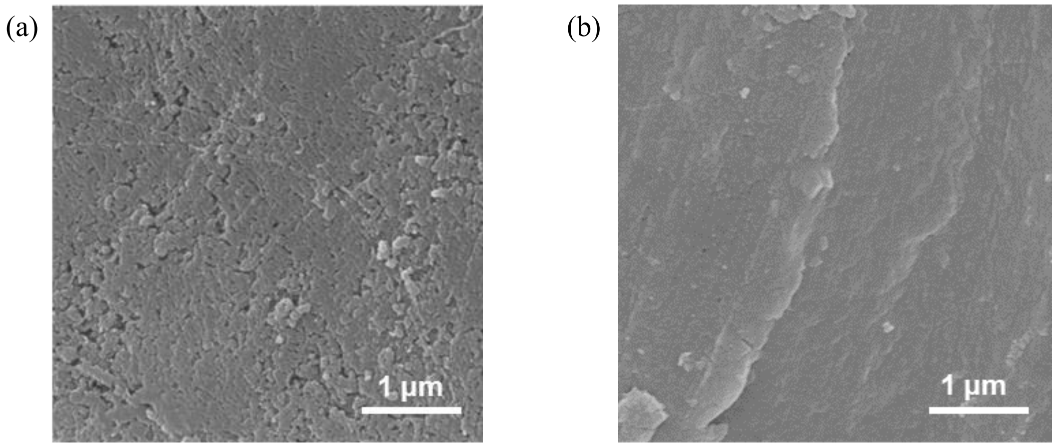

Fig. S2. SEM images of (a) original HPGM electrode and (b) HPGM electrode filled with PAA-based GPE

(a) (b)

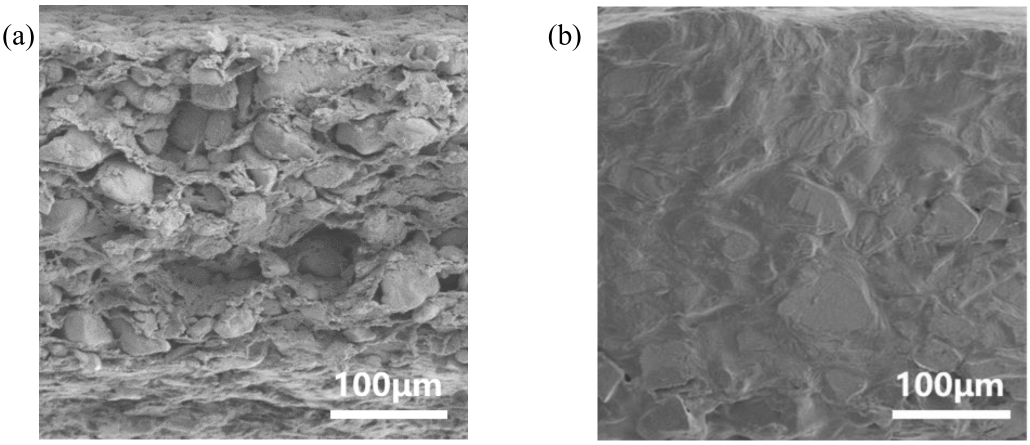

Fig. S3. Cross-sectional SEM images of (a) original HPGM electrode and (b) HPGM electrode filled with PAA-based GPE

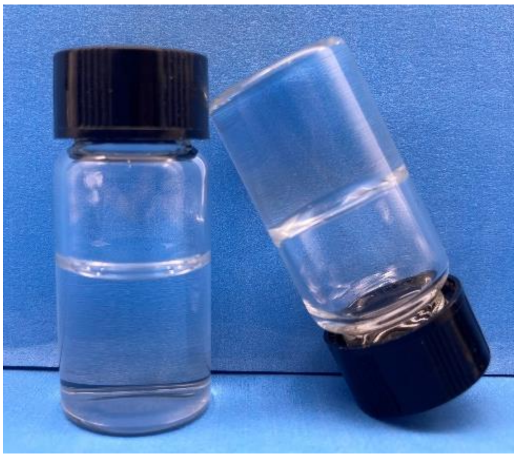

Fig. S4. Optical image of PAA-based GPE before and after polymerization.

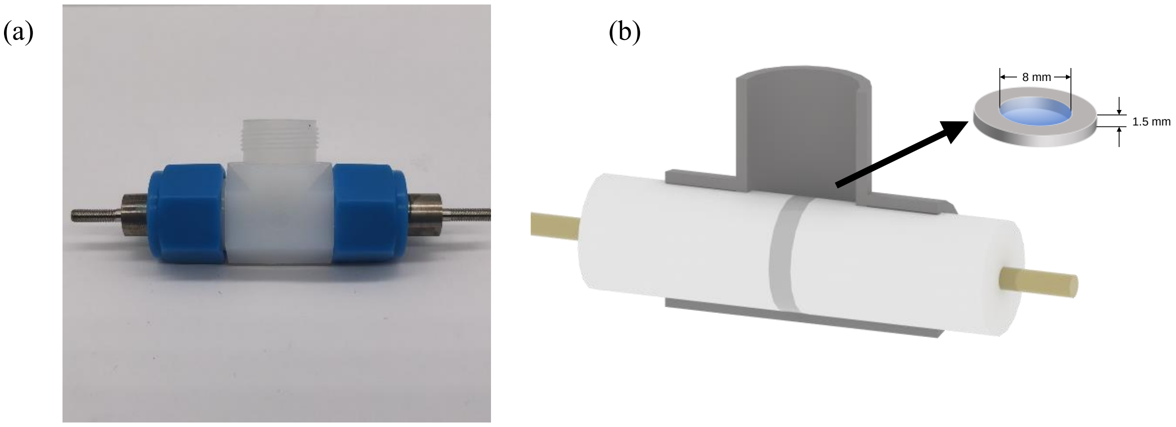

Fig. S5. (a) Optical image of the instrument used for testing ionic conductivity. (b) Diagram of the instrument's internal composition including two electrodes, a rubber ring and the gel polymer electrolyte (blue).

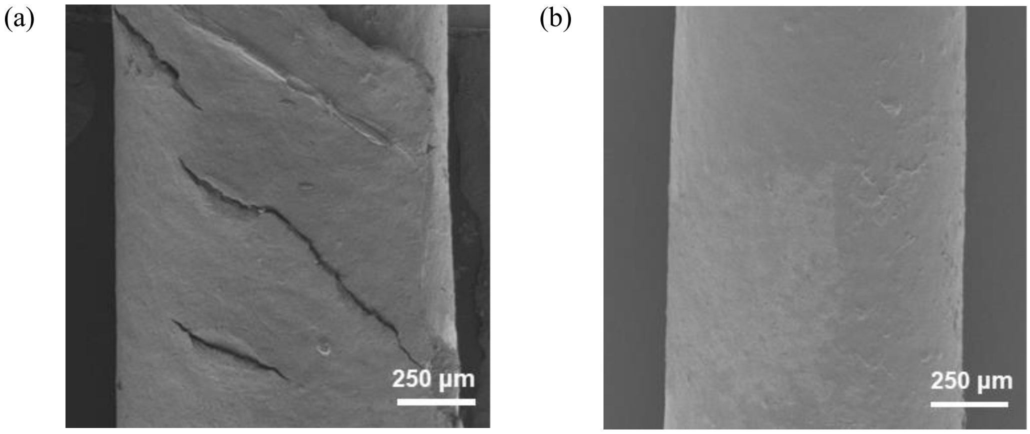

Fig. S6. SEM images of a CNT film which was immersed in different electrolytes after folding several times. (a) 1 M H_2SO_4 solution, (b) PAA-based GPE.

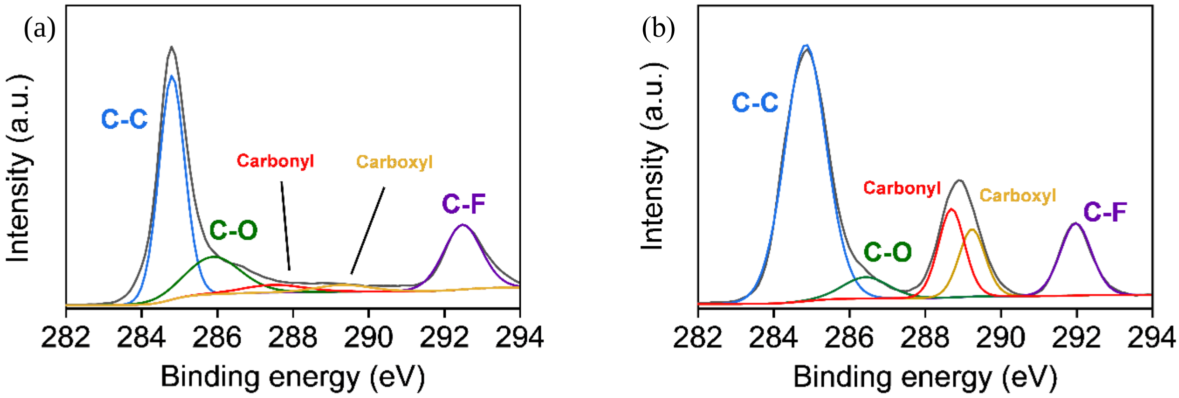

Fig. S7. XPS spectra of the HPGM electrodes treated by different electrolytes. (a) 1 M H_2SO_4 solution, (b) PAA-based GPE.

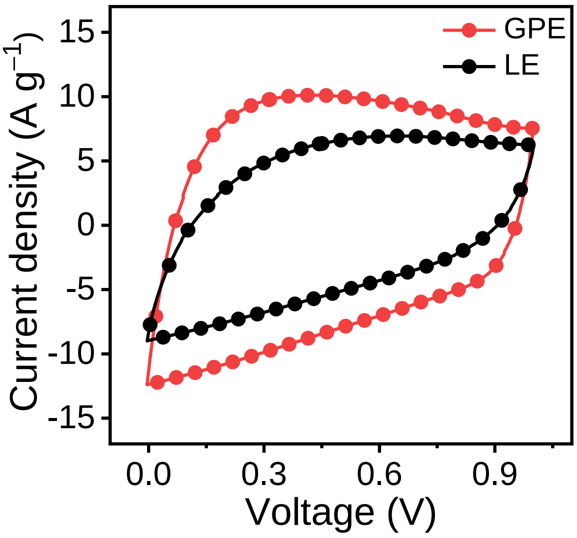

Fig. S8. CV curves of 20 μm-thick-electrodes (mass loading: 2 mg cm^{-2}) with GPE and LE collected at 100 mV s^{-1}.

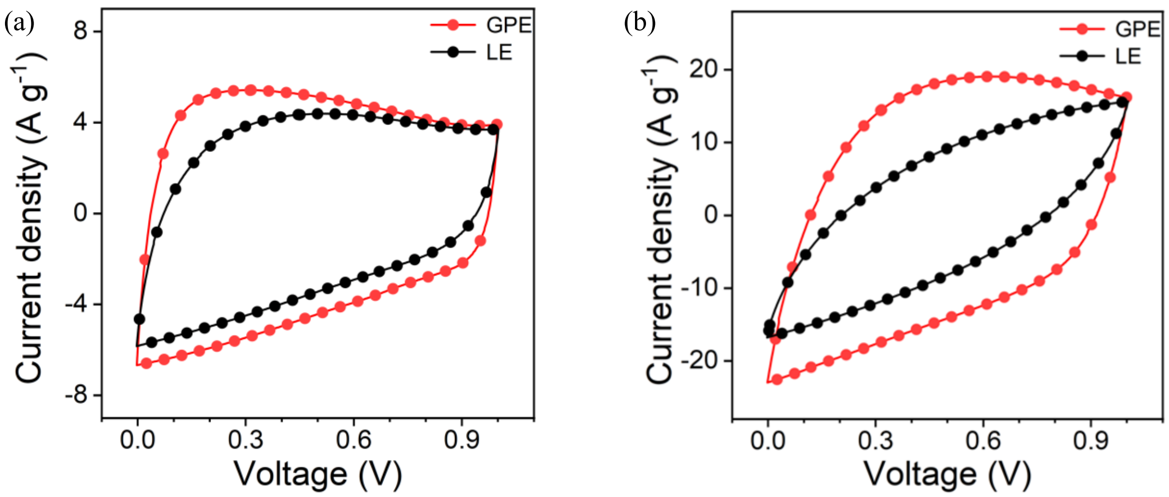

Fig. S9. CV curves of 20 μm-thick-electrodes (mass loading: 2 mg cm^{-2}) with GPE and LE collected at (a) 50 mV s^{-1}, and (b) 200 mV s^{-1}.

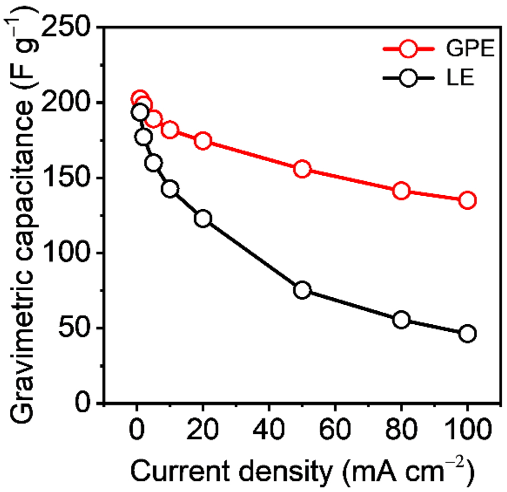

Fig. S10. The rate capability of HPGM electrodes with LE and PAA-based GPE based on gravimetric capacitance.

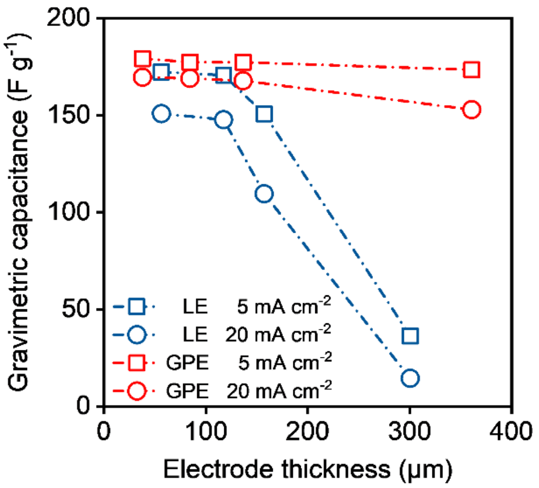

Fig. S11. The gravimetric capacitance of HPGM electrodes with different thicknesses using LE and PAA-based GPE.

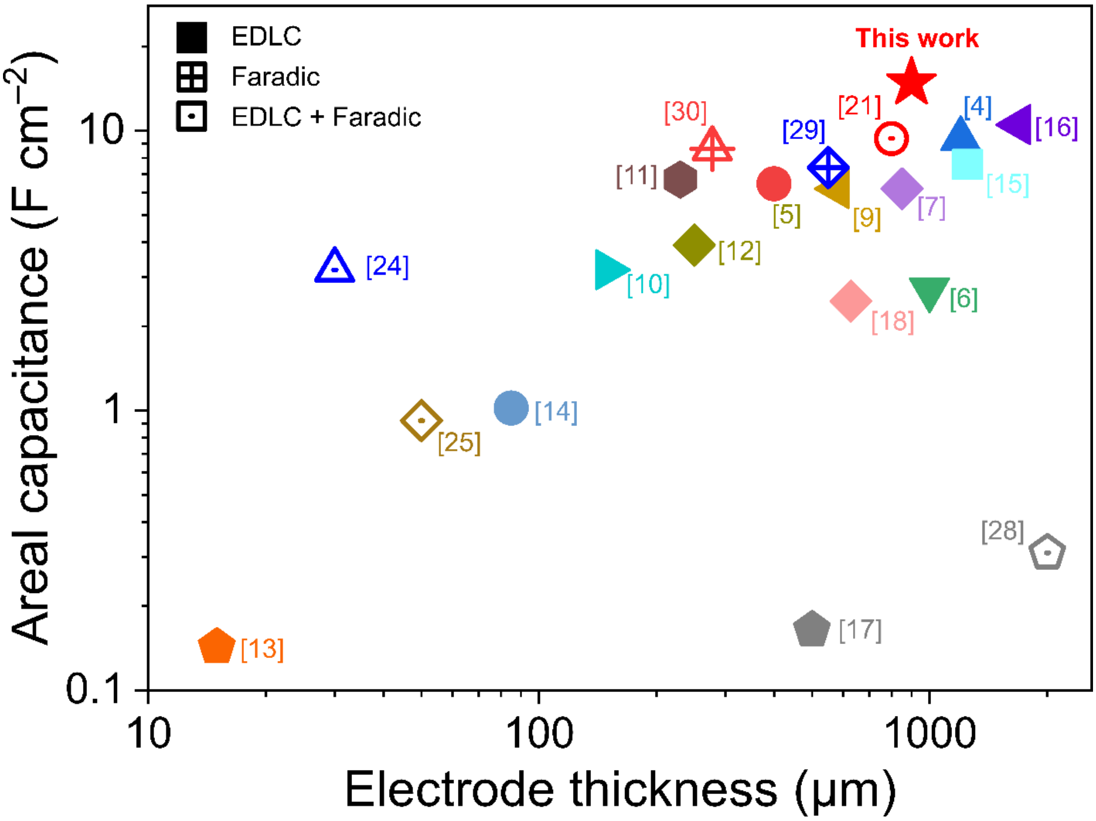

Fig. S12. The comparison of the areal capacitance of electrode with different thicknesses.

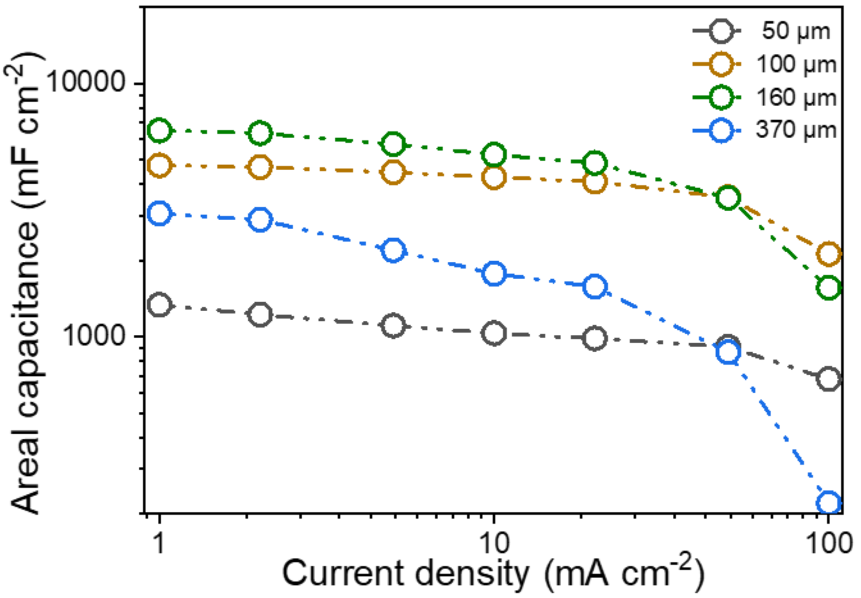

Fig. S13. Rate capabilities of SC with different electrode thicknesses using LE.

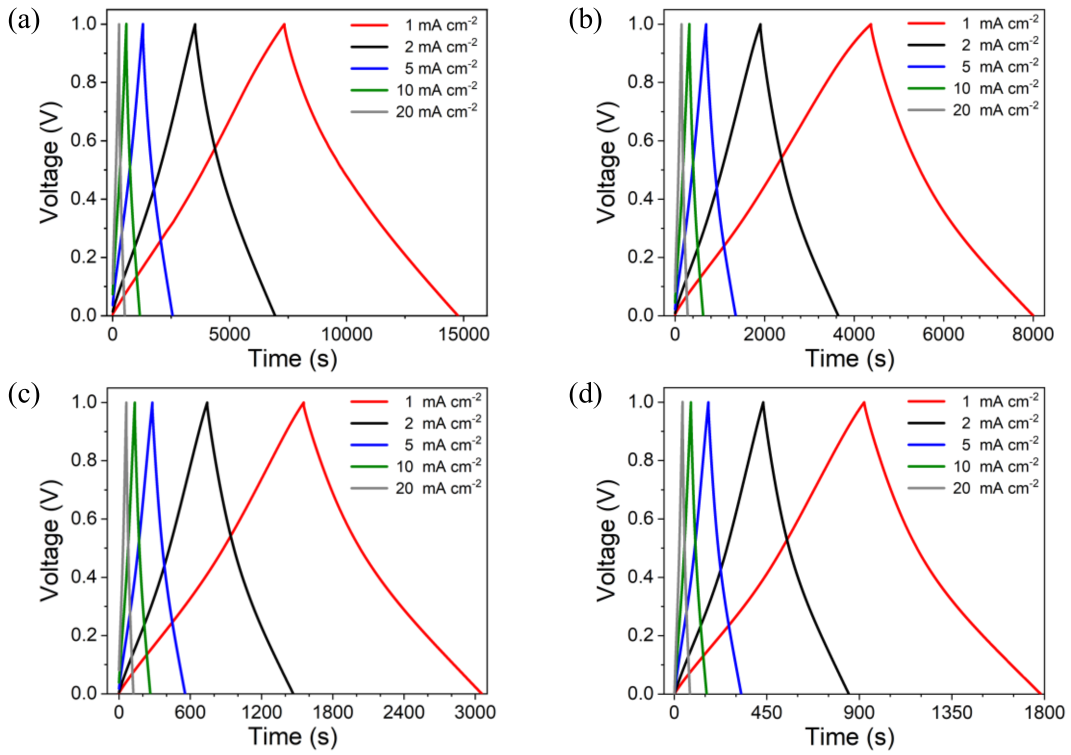

Fig. S14. GCD curves of SC with different electrode thicknesses using PAA-based GPE. (a) 900 μm, (b) 370 μm, (c) 160 μm, (d)100 μm.

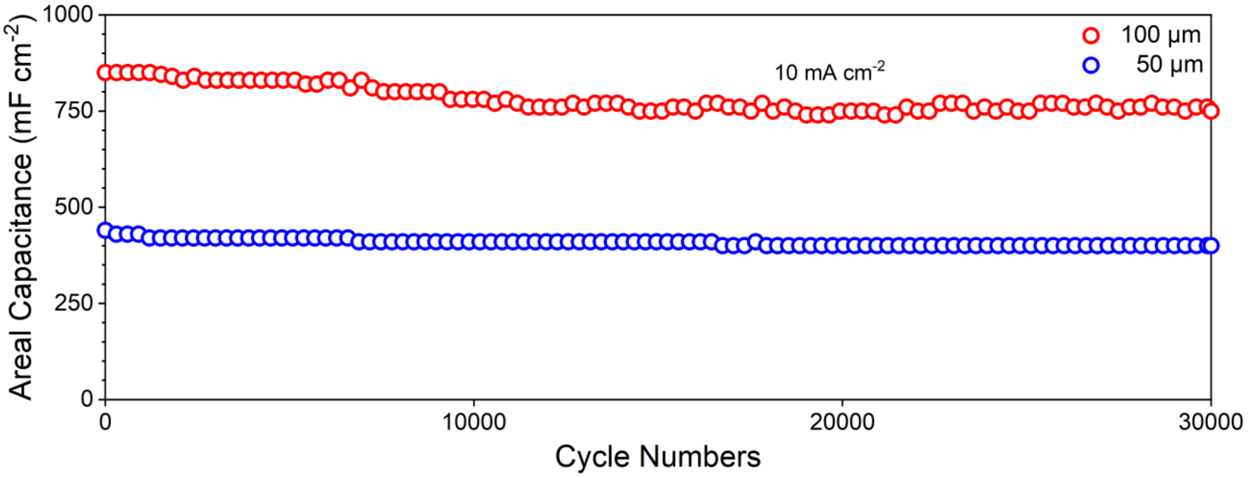

Fig. S15. Cycling stability of the device with different thicknesses of electrode using PAA-based GPE at 10 mA cm^{-2}.

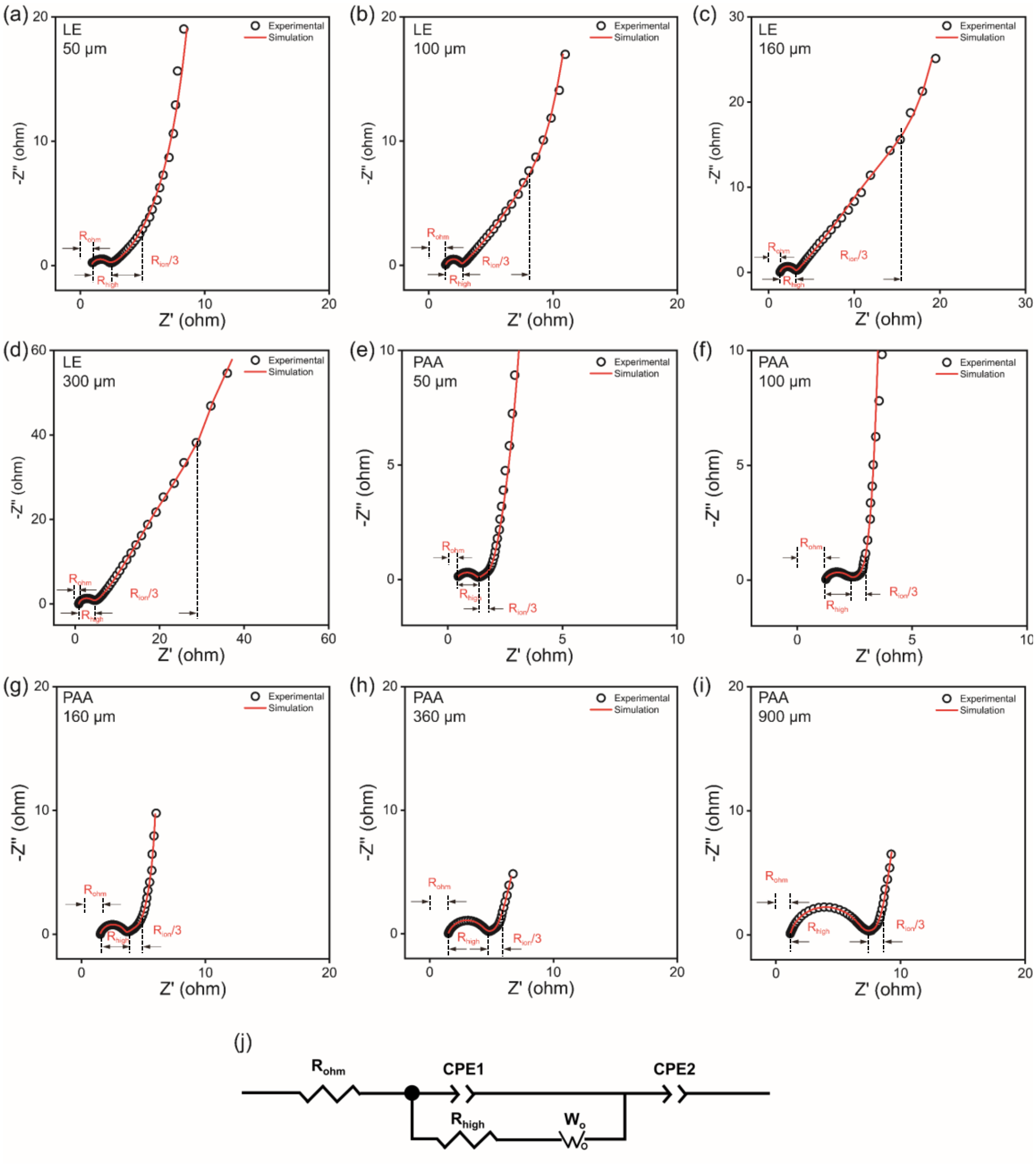

Fig. S16. Nyquist plots and simulation results of SCs assembled using electrodes with a thickness of (a) 50 μm, (b) 100 μm, (c) 160 μm, (d) 300 μm in LE and (e) 50 μm, (f) 100 μm, (g) 160 μm, (h) 360 μm, (i) 900 μm in PAA-based GPE. The solid lines are the best-fitting simulations for the equivalent circuits using the generalized finite length Warburg element open circuit terminus (W_o) as shown in (j)[2].

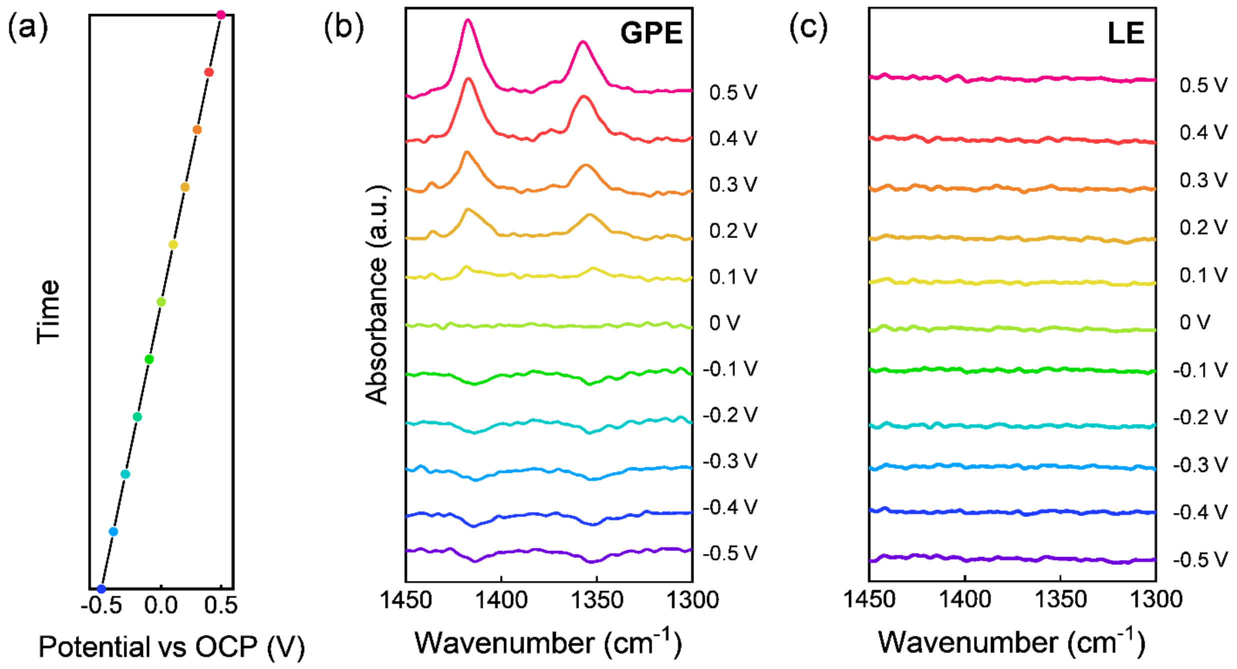

Fig. S17. (a) Galvanostatic charge-discharge curve of HPGM electrodes. *In situ* ATR-SEIRAS spectra of HPGM electrodes with different potentials in electrolytes of (b) PAA-based GPE and (c) LE.

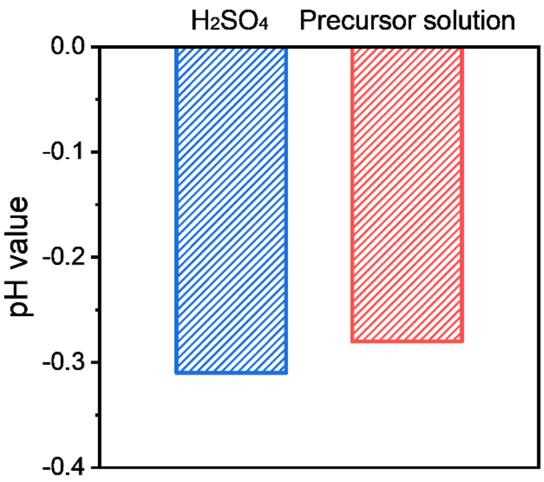

Fig. S18. The pH values of different electrolytes.

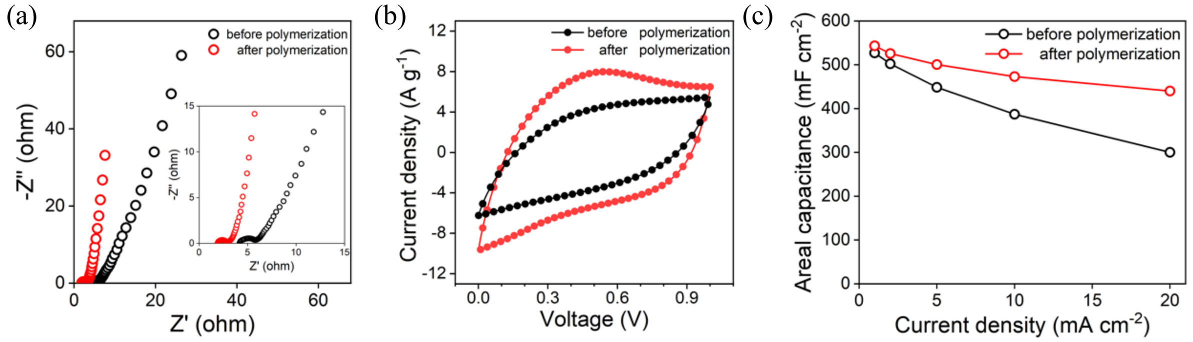

Fig. S19. (a) EIS, and (b) CV curves collected at 100 mV s^{-1}, and (c) rate capability of the electrodes with PAA-based GPE and precursor solution.

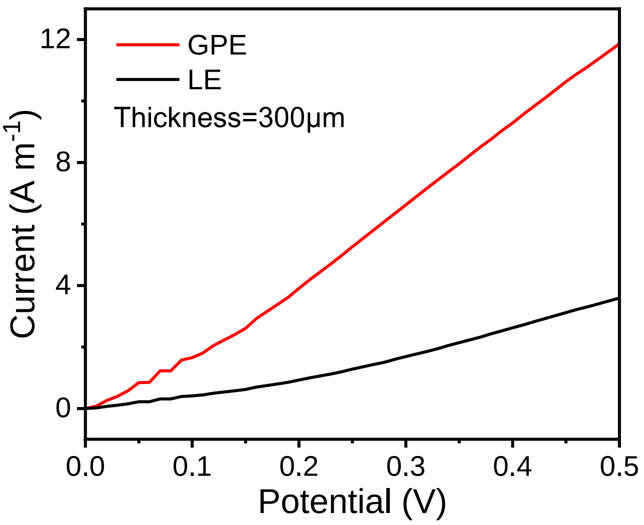

Fig. S20. Current density of porous electrodes obtained by FEM simulations (Potential scan rate is 0.05 V s^{-1}).

Table S2. Comparison of basic properties and capacitive performance of thick electrodes with various electrolytes.

Electrode	Type	Thickness (μm)	Electrolyte	Current density or Scan rate	Volumetric capacitance (F cm^{-3})	Areal capacitance (F cm^{-2})	Ref.
**HPGM**	**EDLC**	**900**	**PAA-based GPE**	**1 mA cm^{-2}**	**165**	**14.85**	**This work**
EEG	EDLC	57.5	PVA/H_2SO_4	1 mA cm^{-2}	0.365	2.1×10^{-3}	[3]
IWBM	EDLC	1200	4 M KOH	50 mA g^{-1}	78.0	9.36	[4]
Graphene pellets	EDLC	400	6 M KOH	5 mV s^{-1}	162	6.48	[5]
WBMs	EDLC	1000	2 M KOH	10 mA cm^{-2}	27.0	2.7	[6]
SLC	EDLC	850	6 M KOH	1 mA cm^{-2}	72.7	6.2	[7]
EGM-rGO(50%EG)	EDLC	NA	$EMIMBF_4$/AN	1 A g^{-1}	203	NA	[8]
MWCNT/rGO	EDLC	580	PVA/LiCl	20 mA cm^{-2}	106.9	6.2	[9]
BNP-HGH	EDLC	150	$EMIMBF_4$/AN	1 A g^{-1}	212	3.18	[10]
F-GRF	EDLC	230	6 M KOH	5 mA cm^{-2}	293	6.7	[11]
PAC-rGO	EDLC	250	6 M KOH	1.2 mA cm^{-2}	156	3.9	[12]
hnp-G	EDLC	15	PVA/H_2SO_4	0.1 A g^{-1}	95.4	0.143	[13]
PPy NT@rGO	EDLC	85	PVA/H_2SO_4	1 mA cm^{-2}	113.8	1.024	[14]
C-ZIF-8@ACW	EDLC	1250	PVA/KOH	1 mA cm^{-2}	60.72	7.59	[15]

3D-CL-A66%	EDLC	1690	1 M $NaSO_4$	3 mA cm^{-2}	62.13	10.5	[16]
MWCNT	EDLC	500	PVA/H_3PO_4	1 mA cm^{-2}	3.3	0.165	[17]
CNTs	EDLC	629	$TEABF_4$/ACN	10 mV s^{-1}	39	2.46	[18]
PCF-C600	EDLC	NA	3 M KOH	0.1 A g^{-1}	NA	2.79	[19]
MA graphene	EDLC	~200	$EMMIBF_4$	1 A g^{-1}	163	6.52	[20]
CW-P-9.24	EDLC+Faradic	800	6 M KOH	1 mA cm^{-2}	117.5	9..4	[21]
P–Mn_3O_4/C	EDLC+Faradic	774	1 M Na_2SO_4	1 mA cm^{-2}	112.9	8.74	[22]
rGOFFs	EDLC+Faradic	220	1 M H_2SO_4	1 mA cm^{-2}	127.8	2.812	[23]
BMX yarn	EDLC+Faradic	30	3 M H_2SO_4	2 mA cm^{-2}	1083	3.188	[24]
GHPO-3	EDLC+Faradic	50	1 M KOH	5 mA cm^{-2}	184	0.92	[25]
ZnCW-1000	EDLC+Faradic	800	5 M H_2SO_4	2 mA cm^{-2}	150	11.96	[26]
Ti_3C_2Tx/WOx@CC	EDLC+Faradic	NA	PVA/H_2SO_4	5 mA cm^{-2}	NA	3.8	[27]
rGO@MnO_x/CNT	EDLC+Faradic	2000	1 M $NaSO_4$	0.5 mA cm^{-2}	1.55	0.31	[28]
TALP	Faradic	278	PVA/PEG	1 mA cm^{-2}	310.5	8.63	[29]
PBA/CNT/KB	Faradic	550	EG60	1 mA cm^{-2}	134.36	7.39	[30]

Table S3. Comparison of basic properties and capacitive performance of supercapacitors with various electrolytes.

Electrode	Electrolyte	Voltage (V)	Current density or Scan rate	Power density (mW cm^{-2})	Energy density (mWh cm^{-2})	Ref.
**HPGM**	**PAA-based GPE**	**0~1**	**1 mA cm^{-2}**	**0.5**	**1.031**	**This work**
EEG	PVA/H_2SO_4	0~1	1 mA cm^{-2}	0.38	3×10^{-4}	[3]
CW-P-9.24	6 M KOH	0~1.2	1 mA cm^{-2}	0.6	0.94	[21]
P–Mn_3O_4/C@AC	NA	0~2.4	1 mA cm^{-2}	1.7	0.573	[22]
TALP	PVA/PEG	0~0.8	1 mA cm^{-2}	NA	0.795	[29]
IWBM	4 M KOH	-0.8~0	50 mA g^{-1}	1.2	0.832	[4]
PEDOT/PSS@MWCNT	PVA/H_3PO_4	0~1.2	2 mV s^{-1}	3.24	0.539	[17]
WBMs	2 M KOH	0~1	10 mA cm^{-2}	4.61	0.375	[6]
GF-Ni-Au@NiO_x	2 M KOH	0~1.6	3 mA cm^{-2}	0.52	0.593	[31]
PANI/rGOFFs	1 M H_2SO_4	0~1.0	1 mA cm^{-2}	0.254	2.8×10^{-2}	[23]
EGM-rGO(50%EG)	$EMIMBF_4$/AN	0~4	15 mA cm^{-2}	NA	NA	[8]
MWCNT/rGO	PVA/LiCl	0~0.8	20 mA cm^{-2}	~1.9	NA	[9]
BPN-HGH	PVA/H_2SO_4	0~1.0	1.5 mA cm^{-2}	NA	0.86	[10]
F-GRF	6 M KOH	-1.0~0	5 mA cm^{-2}	3.77	0.52	[11]
BMX yarn	3 M H_2SO_4	0~1	2 mA cm^{-2}	16.28	0.185	[24]

PAC-rGO	6 M KOH	0~1.5	1.2 mA cm^{-2}	NA	NA	[12]
PPy	PVA/H_2SO_4	-0.2~0.8	5 mV s^{-1}	0.17	3.855×10^{-2}	[32]
hnp-G	PVA/H_2SO_4	0~1	0.1 A g^{-1}	62.4	7.95×10^{-3}	[13]
NRGF	PVA/LiCl	0~2.2	0.5 mA cm^{-2}	NA	3.66×10^{-2}	[33]
MSC-F15	PVA/H_2SO_4	0~1	0.5 mA cm^{-2}	2.12	0.252	[34]
PPy NT@rGO	PVA/H_2SO_4	-0.2~0.8	1 mA cm^{-2}	10	6.14×10^{-2}	[14]
CTA-CNT/AC	1 M H_2SO_4	0~0.9	1 mA cm^{-2}	1.3	0.72	[35]
GHPO-3	1 M KOH	0~1.4	5 mA cm^{-2}	1.05	0.13	[25]
ZnCW-1000	5 M H_2SO_4	0~1.6	2 mA cm^{-2}	2.56	1.62	[26]
C-ZIF-8@ACW	PVA/KOH	0~1	1 mA cm^{-2}	0.5	0.55	[15]
CL-MnO_2@CL-A	1 M $NaSO_4$	0~1.8	3 mA cm^{-2}	2.48	0.808	[16]
CNT-5% CMC	$TEABF_4$/ACN	0~2.7	10 mV s^{-1}	16.61	2.49	[18]
Ti_3C_2Tx/WOx@CC	PVA/H_2SO_4	0~0.6	5 mA cm^{-2}	1.5	9.68×10^{-3}	[27]
CNT/KB@WO_3 and PBA	EG60	0~1.5	0.5 mA cm^{-2}	NA	1.08	[30]
rGO/MnO_x/CNT	1 M $NaSO_4$	0~0.8	0.5 mA cm^{-2}	0.16	2.75×10^{-2}	[28]
PCF-C600	3 M KOH	-1~0	0.1 A g^{-1}	NA	0.388	[19]
NiO/CF@GS and Fe_2O_3	PVA/KOH	0~1.8	3 mA cm^{-2}	30	0.093	[36]

Table S4. Resistance determined from TLM for supercapacitors assembled using electrodes with different thicknesses using LE and PAA-based GPE.

Electrolyte	Electrode thickness (μm)	R_{ohm} (Ω cm^{-2})	R_{ct} (Ω cm^{-2})	R_{ion} (Ω cm^{-2})
LE	50	1.21	1.95	8.03
	100	1.73	1.71	20.56
	160	1.69	2.38	46.92
	300	1.11	4.76	93.94
GPE	50	0.55	1.16	1.82
	100	1.55	1.46	2.48
	160	1.92	2.70	3.86
	360	1.94	4.10	4.59
	900	1.57	7.61	6.38

References:

1. Miyake H, Ye S, Osawa M. Electroless deposition of gold thin films on silicon for surface-enhanced infrared spectroelectrochemistry. *Electrochemistry Communications*. 2002; **4**(12): 973-977.
2. Ogihara N, Itou Y, Sasaki T *et al.* Impedance Spectroscopy Characterization of Porous Electrodes under Different Electrode Thickness Using a Symmetric Cell for High-Performance Lithium-Ion Batteries. *The Journal of Physical Chemistry C*. 2015; **119**(9): 4612-4619.
3. Shi Q, Xiang Y, Ji G *et al.* Flexible Planar-Integrated Micro-Supercapacitors from Electrochemically Exfoliated Graphene as Advanced Electrodes Prepared by Flash Foam–Assisted Stamp Technique on Paper. *Energy Technology*. 2019; **7**(11).
4. Yang L, Takkallapally C, Gabhi RS *et al.* Wood Biochar Monolith-Based Approach to Increasing the Volumetric Energy Density of Supercapacitor. *Industrial & Engineering Chemistry Research*. 2022; **61**(23): 7891-7901.
5. Li H, Tao Y, Zheng X *et al.* Ultra-thick graphene bulk supercapacitor electrodes for compact energy storage. *Energy & Environmental Science*. 2016; **9**(10): 3135.
6. Ma Y, Yao D, Liang H *et al.* Ultra-thick wood biochar monoliths with hierarchically porous structure from cotton rose for electrochemical capacitor electrodes. *Electrochimica Acta*. 2020; **352**: 136452.
7. Li H, Yuan D, Tang C *et al.* Lignin-derived interconnected hierarchical porous carbon monolith with large areal/volumetric capacitances for supercapacitor. *Carbon*. 2016; **100**: 151-157.
8. Li Z, Gadipelli S, Li H *et al.* Tuning the interlayer spacing of graphene laminate films for efficient pore utilization towards compact capacitive energy storage. *Nature Energy*. 2020; **5**(2): 160-168.
9. Yang Y, Huang Q, Niu L *et al.* Waterproof, Ultrahigh Areal-Capacitance, Wearable Supercapacitor Fabrics. *Advanced Materials*. 2017; **29**(19): 1606679.
10. Pan Z, Zhi H, Qiu Y *et al.* Achieving commercial-level mass loading in ternary-doped holey graphene hydrogel electrodes for ultrahigh energy density supercapacitors. *Nano Energy*. 2018; **46**: 266-276.
11. Sheng L, Chang J, Jiang L *et al.* Multilayer-Folded Graphene Ribbon Film with Ultrahigh Areal Capacitance and High Rate Performance for Compressible Supercapacitors. *Advanced Functional Materials*. 2018; **28**(21).
12. Choi J-H, Kim Y, Kim B-s. Multifunctional role of reduced graphene oxide binder for high performance supercapacitor with commercial-level mass loading. *Journal of Power Sources*. 2020; **454**: 227917.
13. Qin K, Kang J, Li J *et al.* Continuously hierarchical nanoporous graphene film for flexible solid-state supercapacitors with excellent performance. *Nano Energy*. 2016; **24**: 158-164.
14. Yang C, Zhang L, Hu N *et al.* Reduced graphene oxide/polypyrrole nanotube papers for flexible all-solid-state supercapacitors with excellent rate capability and high energy density. *Journal of Power Sources*. 2016; **302**: 39-45.
15. Zhang WJ, Li M, Zhong L *et al.* A family of MOFs@Wood-Derived hierarchical porous composites as freestanding thick electrodes of solid supercapacitors with enhanced areal capacitances and energy densities. *Materials Today Energy*. 2022; **24**: 100951.
16. Katsuyama Y, Haba N, Kobayashi H *et al.* Macro- and Nano-Porous 3D-Hierarchical Carbon Lattices for Extraordinarily High Capacitance Supercapacitors. *Advanced Functional Materials*. 2022; **32**(24): 2201544.
17. Li X, Shao J, Kim SK *et al.* High energy flexible supercapacitors formed via bottom-up infilling of gel electrolytes into thick porous electrodes. *Nature Communications*. 2018; **9**(1): 2578.
18. Pokhriyal A, González-Gil RM, Bengoa LN *et al.* Nanostructured Thick Electrode Strategies toward Enhanced Electrode–Electrolyte Interfaces. *Materials*. 2023; **16**(9): 3439.
19. Bai L, Wang C, Bai L *et al.* Cattails-derived porous carbon fibers for high mass loading supercapacitors. *Journal of Porous Materials*. 2022; **30**(2): 579-587.

20. Li C, Li X, Yang Q *et al.* Vascular System Inspired 3D Electrolyte Network for High Rate and High Mass Loading Graphene Supercapacitor. *Advanced Functional Materials*. 2024: 2315137.
21. Wang F, Cheong JY, He Q *et al.* Phosphorus-doped thick carbon electrode for high-energy density and long-life supercapacitors. *Chemical Engineering Journal*. 2021; **414**: 128767.
22. Guo W, Yu C, Zhao C *et al.* Boosting charge storage in 1D manganese oxide-carbon composite by phosphorus-assisted structural modification for supercapacitor applications. *Energy Storage Materials*. 2020; **31**: 172-180.
23. Shao F, Hu N, Su Y *et al.* Non-woven fabric electrodes based on graphene-based fibers for areal-energy-dense flexible solid-state supercapacitors. *Chemical Engineering Journal*. 2020; **392**: 123692.
24. Wang Z, Qin S, Seyedin S *et al.* High-Performance Biscrolled MXene/Carbon Nanotube Yarn Supercapacitors. *Small*. 2018; **14**(37): 1802225.
25. Wang J, Zhang S, Sun J *et al.* Gradient porous electrode with high mass loading derived by ultrafast self-combustion for supercapacitor and oxygen evolution reaction. *Journal of Energy Storage*. 2023; **74**: 109409.
26. Yan B, Zhao W, Zhang Q *et al.* One stone for four birds: A “chemical blowing” strategy to synthesis wood-derived carbon monoliths for high-mass loading capacitive energy storage in low temperature. *Journal of Colloid and Interface Science*. 2024; **653**: 1526-1538.
27. Pan Z, Yang C, Chen Z *et al.* Construction of $Ti_3C_2T_x/WO_x$ heterostructures on carbon cloth for ultrahigh-mass loading flexible supercapacitor. *Nano Research*. 2022; **15**(10): 8991-8999.
28. Gao Y, Ding J. 3D Printed Thick Reduced Graphene Oxide: Manganese Oxide/Carbon Nanotube Hybrid Electrode with Highly Ordered Microstructures for Supercapacitors. *Advanced Materials Technologies*. 2022; **8**(2): 2200263.
29. Xiao K, Yang T, Liang J *et al.* Nanofluidic voidless electrode for electrochemical capacitance enhancement in gel electrolyte. *Nature Communications*. 2021; **12**(1): 5515.
30. Zhang M, Xu T, Wang D *et al.* A 3D-Printed Proton Pseudocapacitor with Ultrahigh Mass Loading and Areal Energy Density for Fast Energy Storage at Low Temperature. *Advanced Materials*. 2023; **35**(23): 2209963.
31. Wang Y, Su S, Cai L *et al.* Hierarchical supercapacitor electrodes based on metallized glass fiber for ultrahigh areal capacitance. *Energy Storage Materials*. 2019; **20**: 315-323.
32. Zhang J, Chen Q, Zhang H *et al.* High-Performance Polypyrrole Coated Filter Paper Electrode for Flexible All-Solid-State Supercapacitor. *Journal of The Electrochemical Society*. 2020; **167**(14): 140533.
33. Liu Q, Zhou J, Song C *et al.* 2.2V high performance symmetrical fiber-shaped aqueous supercapacitors enabled by “water-in-salt” gel electrolyte and N-Doped graphene fiber. *Energy Storage Materials*. 2020; **24**: 495-503.
34. Cai J, Lv C, Hu C *et al.* Laser direct writing of heteroatom-doped porous carbon for high-performance micro-supercapacitors. *Energy Storage Materials*. 2020; **25**: 404-415.
35. Yuan R, Sun S, Ling S *et al.* Solvent exchange assisted 3D printing of low tortuosity thick electrode for high areal energy density and power density supercapacitors. *Carbon*. 2024; **218**: 118737.
36. Zeng F, Song XY, Liang J *et al.* Multi-electron/ion conduction channels enabling high-performance flexible supercapacitors. *Journal of Materials Chemistry A*. 2022; **10**(47): 25148-25158.
